# Supplementary material for: Biogeochemical processes create distinct isotopic fingerprints to track floodplain rearing of juvenile salmon
Source: PLoS One. 2021 Oct 28;16(10):e0257444. doi: 10.1371/journal.pone.0257444 (PMC8553044; doi:10.1371/journal.pone.0257444)
Supplement: S1 File — (DOCX) [file pone.0257444.s001.docx]

# Supplementary Information

## Supplemental A: Fish Collection

### 1999

Juvenile salmon from the Yolo Bypass were collected with methods established by Sommer et al., [31].These fish were collected weekly around the perimeter of the Yolo Bypass from January through April using a 15m beach seine (4.75mm mesh).

### 2014

Nine 0.79-ha replicated experimental fields were built on the Knaggs Ranch, a rice farm located within the Yolo Bypass. All fields shared a common layout and same water sources which was fed from a supply canal through a 60 cm diameter “rice box” or a plastic culvert equipped with a face-mounted slot into which 3.8 x 13.8cm flashboards were placed – at the fields’ northwest corner. Fields were drained through a similar rice box at the southeast corner. The number of boards placed in the slot on the face of the inlet rice box controlled volume of inflow. Surface water elevation in the field was controlled by flashboard elevation at the outlet. Inlet and outlet rice boxes were screened with 3mm plastic mesh in order to pass water but to contain fish. A 150cm x 91cm x 91cm live car made of 3mm plastic mesh was placed in the exit ditch and at the outlet of each field to capture fish that were leaving the fields upon draining. Water elevations over the crown of the fields varied from 0.3 to 0.5m depending on wind and flow conditions.

All of the fields were stomped following harvest of the rice in fall of 2013. Stomping is a farming practice where fields are re-flooded after harvest and machinery is used to incorporate rice straw into the ground in order to promote decomposition. On February 4, 2014, approximately 42,600 fish were delivered by tanker truck from the Feather River Hatchery and enumerated by weight (1104 fish/kg). An average of 4,748 fish were placed into each of the nine experimental fields.

Three fields were chosen at random for sampling. Once per week, fields 2, 6, and 9 were beach seined to sample fish for diet and isotopic analysis. Ten fish were measured, weighed, euthanized and placed into a cooler containing ice before being transferred to UC Davis where they were frozen and stored for diet content and isotope analysis. The reported growth rates from the 2014 field season was derived from the slope of a linear model at 1.06mm/day. The weight growth rate over the same period was 0.15g/day.

### 2015

All of the fields were stomped following harvest of the rice in fall 2014. All of the fields had a 0.5-meter-deep ditch running down the east and south sides of the fields connecting the inlet and outlet control structures. Each ditch was 1.0m wide and dug one meter towards the interior of the field so that depth refuges were outside the striking distance of wading birds such as herons and egrets that frequent the shallow water of the perimeter levees.

On February 5, 2015, approximately 36,056 fish were delivered by tanker truck from the Feather River Hatchery and enumerated by weight (687 fish/kg). An average of 4,006 fish were placed into each of the nine experimental fields. In 2015, California was experiencing a severe drought. This caused the experiment to be ended early on February 27, due to increasing temperatures that could have been potentially lethal.

Fields 1, 4, and 9 were seined to sample fish for diet content and isotopic analysis. Ten fish were measured, weighed, euthanized and placed into a cooler containing ice before being transferred to UC Davis where they were frozen and stored for diet and isotopic analysis. The reported growth rates during the 2015 field season were again derived from the slope of a linear model and the fork length and weight were 0.915 mm/day and 0.123 g/day respectively.

### 2016

In 2016, all of the fields were left fallow due to lack of water allocation to the farm during the historic drought. Tall (>1m) and woody fallow vegetation covered the fields. All of the fields had a 0.5-meter-deep ditch running down the east and south sides of the fields connecting the inlet and outlet control structures that were 1.0m wide and dug one meter towards the interior of the field.

On February 1, 2016, approximately 5,866 fish were delivered by tanker truck from the Feather River Hatchery and enumerated individually. Six of the nine fields were randomly chosen to receive approximately 1,000 fish. One stocked field was drained per week and the first 30 fish were collected from the outlet in a 1.2 × 1.2 × 0.6m live car made of 3mm plastic mesh affixed to the outlet rice box. The draining procedure consisted of removing the inlet screen and placing boards in the inlet rice box, and removing boards from the outlet rice box in an effort to drain the field to the level of the ditch before noon to reduce solar influx and subsequently overheating in a shallow field. Additionally, starting with week 2 through 6 we covered the inlet boards with plastic to decrease flow through the boards which was enticing fish to stay near the inlet during draining.

The first 30 fish that were collected from the live cars were euthanized and placed immediately on ice and transported back to the lab where they transferred to a freezer awaiting diet content and isotopic analysis. The remaining fish were allowed to continue down the exit ditch, which ultimately connects to the Sacramento-San Joaquin Delta. The reported growth rates during the 2016 field season were again derived from the slope of a linear model and the fork length and weight were 1.28mm/day and 0.21g/day respectively.

On March 12, 2016, a small overtopping event over the Fremont weir occurred causing a small portion of the Yolo Bypass to become flooded. This allowed for the opportunity to collect naturally recruited juvenile Chinook Salmon captured within the live cars affixed to the outlet rice boxes while the fields at Knaggs Ranch were draining.

Fin from fish reared and captured weekly on the floodplain were prepared using methods established by Heady and Moore [17] and measured for δ¹³C, δ¹⁵N, δ³⁴S (Supplemental Table S3)

### 2017

In 2017, California experienced the wettest year on record. The replicated fields at Knaggs Ranch used in previous years were inaccessible due to high flows and water depths. Fish collection instead was similar to the methods detailed in Sommer et al., [31], where fish were seined around the perimeter of the Yolo Bypass.

In addition to beach seined fish, 10 fish were placed into each of the three 0.6 × 0.6 × 1.2m enclosures at Knaggs Ranch. The frames of the enclosures were constructed from 19mm polyvinyl chloride (PVC) pipe with 6.3mm extruded plastic netting fitted around the frame. The netting was held in place by plastic cable ties placed at regular intervals to keep the netting close to the frame and each cage was attached to a t-post in the field. These fish were received from the Feather River Hatchery and placed in cages from March 16 – April 28, 2017.

### Agriculture Drainage Canal

The toe drain represents a perennial riparian channel located along the eastern edge of the Yolo Bypass. Water is able to drain out of the Yolo Bypass through the toe drain. Located at the base of the toe drain is a rotary screw trap that is operated January – June. During 2017, the Department of Water Resources collected 15 juvenile Chinook Salmon from this rotary screw trap to identify floodplain use in fish that naturally recruited to the Yolo Bypass during this high flood year.

### Sacramento River

Fall and late-fall run sized fish were collected by the Delta Juvenile Fish Monitoring Program (DJFMP) from sites upstream (e.g. Clarksburg, Sherwood Harbor), within and downstream (Chipps Island) of the Delta during the 2014-15 drought and water year 2016. Fall and late fall run juvenile Chinook Salmon were collected by DJFMP during the winter and spring rearing season (Dec/Jan to June) of 2014-17. The samples were collected by 15m beach seine, midwater trawl, and Kodiak trawl, kept on ice, and frozen until dissections were conducted by UC Davis. Fish for dissections and isotopic analysis were selected from the sites based on their close proximity to the Yolo Bypass.

In 2016 and 2017, 10 fish were placed into each of the three 0.6 × 0.6 × 1.2m enclosures in the Sacramento River. The frames of the enclosures are constructed from 19mm polyvinyl chloride (PVC) pipe with 6.3mm extruded plastic netting fitted around the frame. The netting is held in place by plastic cable ties placed at regular intervals to keep the netting close to the frame and each cage was attached to a t-post in the field. These fish were received from the Feather River Hatchery and placed in cages from March 29, 2016 – April 28, 2016 and March 16 – April 28, 2017.

## Supplemental Table S1

Summary statistics of the stable isotope analyses for stomach contents and muscle tissues from all years sampled (1999, 2012-2017) from the river and floodplain sites. Enclosed sites include fish from cage and experimental fields.

| **Habitat** | **Site** | **Year** | ***Tissue*** | ***δ¹³C mean*** | ***δ¹³C SD*** | ***δ¹³C n*** | ***δ^15^N mean*** | ***δ^15^N SD*** | ***δ^15^N n*** | ***δ³⁴S mean*** | ***δ³⁴S SD*** | ***δ³⁴S n*** |
| --- | --- | --- | --- | --- | --- | --- | --- | --- | --- | --- | --- | --- |
| **River** | Sacramento River | 2012 | M | NA | NA | NA | NA | NA | NA | 5.18 | 5.21 | 8 |
|  | Sacramento River | 2012 | S | NA | NA | NA | NA | NA | NA | 3.29 | 2.14 | 11 |
|  | Sacramento River | 2013 | M | NA | NA | NA | NA | NA | NA | 2.18 | 2.03 | 5 |
|  | Sacramento River | 2013 | S | NA | NA | NA | NA | NA | NA | 0.98 | 1.18 | 6 |
|  | Sacramento River | 2014 | M | -23.28 | 2.75 | 35 | 12.01 | 1.62 | 35 | 6.57 | 4.12 | 38 |
|  | Sacramento River | 2014 | S | -28.35 | 1.5 | 33 | 7.11 | 1.52 | 33 | 2.59 | 2.63 | 36 |
|  | Sacramento River | 2015 | M | -22.44 | 2.27 | 42 | 11.89 | 1.7 | 42 | 4.97 | 4.71 | 42 |
|  | Sacramento River | 2015 | S | -28.21 | 2.58 | 42 | 7.19 | 1.88 | 42 | 1.46 | 2.27 | 42 |
|  | Sacramento River | 2016 | M | -22.14 | 3.45 | 52 | 12.53 | 2.88 | 52 | 8.01 | 5.66 | 52 |
|  | Sacramento River | 2016 | S | -27.91 | 1.62 | 54 | 7.36 | 1.1 | 54 | 2.65 | 2.61 | 47 |
|  | Sacramento River | 2017 | M | -22.99 | 2.51 | 51 | 11.67 | 1.71 | 51 | 7.84 | 4.14 | 51 |
|  | Sacramento River | 2017 | S | -28.83 | 1.35 | 50 | 7.06 | 1.36 | 50 | 2.28 | 1.64 | 50 |
|  | Sacramento River Enclosed | 2015 | M | -21.19 | NA | 1 | 9.29 | NA | 1 | -1.6 | NA | 1 |
|  | Sacramento River Enclosed | 2015 | S | -27.99 | NA | 1 | 10.18 | NA | 1 | -0.8 | NA | 1 |
|  | Sacramento River Enclosed | 2016 | M | -23.12 | 0.81 | 5 | 11.49 | 0.35 | 5 | 7.84 | 1.46 | 5 |
|  | Sacramento River Enclosed | 2016 | S | -28.03 | 1.8 | 5 | 7.53 | 0.88 | 5 | 1.84 | 0.99 | 5 |
|  | Sacramento River Enclosed | 2017 | M | -24.23 | 0.67 | 5 | 12.28 | 0.32 | 5 | 6.88 | 1.15 | 5 |
|  | Sacramento River Enclosed | 2017 | S | -30.65 | 0.5 | 5 | 8.54 | 1.03 | 5 | 2.03 | 0.41 | 5 |
| **Floodplain** | Yolo Bypass | 1999 | M | NA | NA | NA | NA | NA | NA | -1.26 | 2.96 | 32 |
|  | Yolo Bypass | 1999 | S | NA | NA | NA | NA | NA | NA | -0.7 | NA | 1 |
|  | Yolo Bypass | 2014 | M | -21.47 | 1.71 | 3 | 10.47 | 1.31 | 3 | 13.36 | 0.58 | 3 |
|  | Yolo Bypass | 2015 | M | -21.49 | 1.66 | 3 | 10.43 | 1.33 | 3 | 13.38 | 0.58 | 3 |
|  | Yolo Bypass | 2016 | M | -27.75 | 2.99 | 24 | 10.33 | 1.15 | 24 | 0.15 | 5.38 | 24 |
|  | Yolo Bypass | 2016 | S | -30.76 | 0.74 | 13 | 7.74 | 1.28 | 13 | -1.49 | 1.45 | 13 |
|  | Yolo Bypass | 2017 | M | -26.68 | 2.79 | 27 | 11.82 | 0.71 | 27 | 1.24 | 3.82 | 27 |
|  | Yolo Bypass | 2017 | S | -32.17 | 3.17 | 27 | 7.64 | 0.82 | 27 | -1.91 | 1.14 | 27 |
|  | Yolo Bypass Enclosed | 2014 | M | -29.13 | 2.02 | 29 | 11.4 | 0.45 | 29 | -1.03 | 3.31 | 29 |
|  | Yolo Bypass Enclosed | 2014 | S | -33.65 | 1.87 | 28 | 6.69 | 1.05 | 28 | -3.15 | 1.86 | 28 |
|  | Yolo Bypass Enclosed | 2015 | M | -28.8 | 1.44 | 28 | 10.95 | 0.47 | 28 | -0.18 | 2.97 | 27 |
|  | Yolo Bypass Enclosed | 2015 | S | -33.41 | 1.68 | 27 | 6.34 | 0.9 | 27 | -5.93 | 2.51 | 27 |
|  | Yolo Bypass Enclosed | 2016 | M | -31.44 | 3 | 33 | 11.87 | 0.78 | 33 | -1.27 | 3.99 | 33 |
|  | Yolo Bypass Enclosed | 2016 | S | -34.49 | 2.62 | 32 | 7.68 | 1.41 | 32 | -4.11 | 1.3 | 32 |
|  | Yolo Bypass Enclosed | 2017 | M | -25.98 | 0.8 | 5 | 12.46 | 0.27 | 5 | 2.68 | 1.13 | 5 |
|  | Yolo Bypass Enclosed | 2017 | S | -28.41 | 0.5 | 5 | 7.45 | 0.14 | 5 | -1.96 | 0.46 | 5 |

## Supplemental Table S2

Summary of mixed effect models for δ³⁴S and δ^13^C from stomach contents.

| **Model** | **Variable** | **Variance** | **Std. Dev** | **Std. Error** | **t value** | **Effect Type** |
| --- | --- | --- | --- | --- | --- | --- |
| δ³⁴S Stomach Contents Model | Subsite | 0.8604 | 0.9276 |  |  | Random |
| δ³⁴S Stomach Contents Model | Water Year Type | 0.1078 | 0.3283 |  |  | Random |
| δ³⁴S Stomach Contents Model | Intercept | -2.4491 |  | 0.5302 | -4.62 | Fixed |
| δ³⁴S Stomach Contents Model | Site (river vs floodplain) | 4.7447 |  | 1.0646 | 4.457 | Fixed |
|  | Number of obs: 314, groups: subsite, 7; WDN, 3; Conditional R^2^ 0.57 | | | | |  |
| δ¹³C Stomach Contents Model | Subsite | 5.5903 | 2.3644 |  |  | Random |
| δ¹³C Stomach Contents Model | Water Year Type | 0.1495 | 0.3867 |  |  | Random |
| δ¹³C Stomach Contents Model | Intercept | -32.238 |  | 1.112 | -29.001 | Fixed |
| δ¹³C Stomach Contents Model | Site (river vs floodplain) | 3.868 |  | 2.607 | 1.484 | Fixed |
|  | Number of obs: 300, groups: subsite, 6; WDN, 3; Conditional R^2^ 0.71 | | | | |  |

## Supplemental Table S3

Summary statistics of the stable isotope analyses for fin tissues from all years sampled (1999, 2016) from the Yolo Bypass and Yolo Bypass Enclosed sites.

| **Site** | **Year** | ***Tissue*** | ***δ¹³C mean*** | ***δ¹³C SD*** | ***δ¹³C n*** | ***δ^15^N mean*** | ***δ^15^N SD*** | ***δ^15^N n*** | ***δ³⁴S mean*** | ***δ³⁴S SD*** | ***δ³⁴S n*** |
| --- | --- | --- | --- | --- | --- | --- | --- | --- | --- | --- | --- |
| Yolo Bypass | 1999 | F | NA | NA | NA | NA | NA | NA | -0.95 | NA | 1 |
| Yolo Bypass | 2016 | F | -28.92 | 4.24 | 11 | 11.95 | 1.08 | 11 | 1.48 | 5.54 | 11 |
| Yolo Bypass Enclosed | 2016 | F | -31.82 | 2.49 | 19 | 11.28 | 0.77 | 19 | -2.78 | 3.46 | 19 |

## Supplemental B: Sulfur Isoscape

In order to determine how prey from the Yolo Bypass and Sacramento River compared isotopically to prey in other potential juvenile salmon rearing habitats, a ubiquitous amphipod, *Gammarus sp*., was used as a consumer proxy. Amphipods were collected from the Yolo Bypass as well as other tidal wetland habitats that could potentially display similar low sulfur values (Fig 1, Supplemental Fig S1). Amphipods were collected using 25cm x 30cm D-frame net with 500-micron mesh for all sweep net samples. Approximately 5-15 amphipods from each sample site were homogenized and placed into 8 x 5mm tin capsule to meet the 2-5mg weight requirement for combined δ¹³C, δ¹⁵N, δ³⁴S isotopic analysis. Each capsule was placed in 96-well plate and dried for 48 hours in a drying oven with temperatures not exceeding 55°C. Tins were then crimped and folded into small discs, then placed back into the 96-well plate. Isotopic values of amphipods collected on the Yolo Bypass were compared to the gut contents of the caged salmon on the Yolo Bypass to test the feasibility of using amphipods as salmon food web indicators. Amphipod isotope values were spatially displayed using Arc GIS to illustrate landscape-level variation in isotopic values and infer the extent to which other habitats had overlapping values to the Yolo Bypass (Supplemental Fig S1). Amphipods collected from each location showed a high degree of heterogeneity in δ³⁴S values throughout the landscape (Supplemental Fig S1). However, too few individual amphipods were collected at each sampling location to provide replicates for a formal statistical comparison within site. The Yolo Bypass was the only location sampled with a δ³⁴S value below 0 ‰. The δ³⁴S value measured in amphipods seen on the Yolo Bypass (δ³⁴S = -2.2‰) was consistent with lower values observed within stomach contents from fish reared in this location.

**
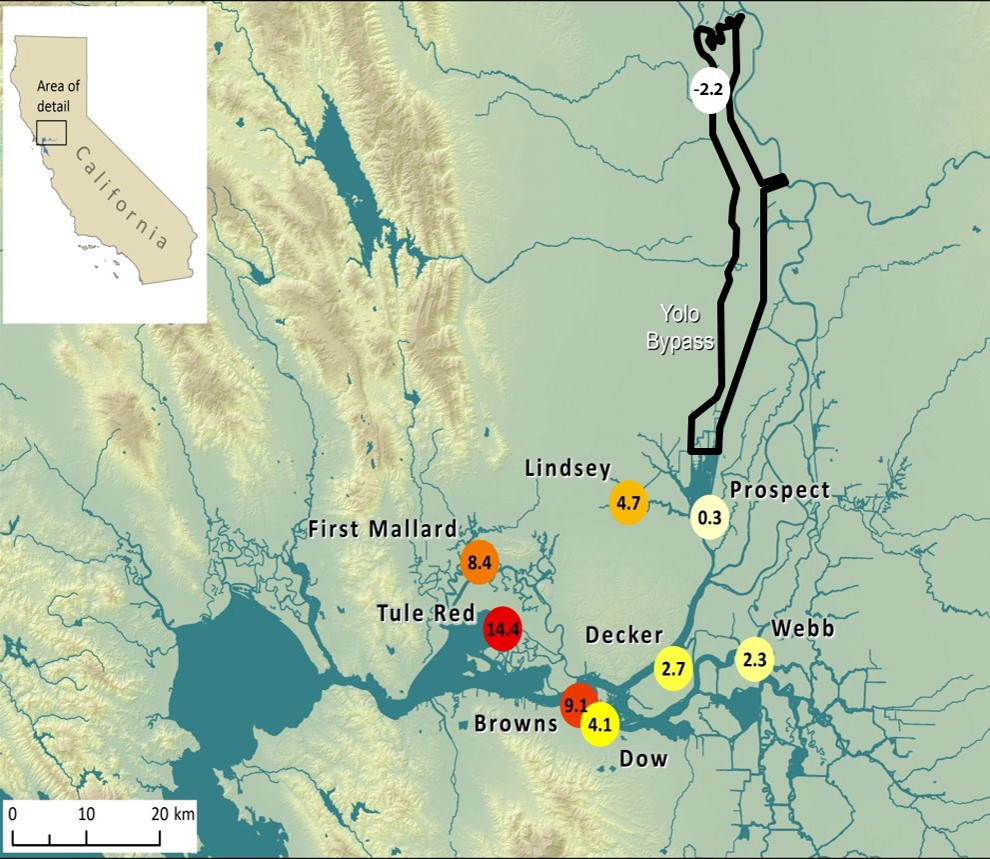
**

## Supplemental Fig S1

Sulfur isoscape of the Lower Sacramento River and Sacramento-San Joaquin Delta. Amphipod species collected at a variety of locations and times within the Central Valley of California provide a snapshot of δ³⁴S values throughout the Central Valley landscape. This isoscape displays how δ³⁴S values increase from freshwater to saltwater with white representing lower values to red representing higher values. The Yolo Bypass was the only location sampled that had a δ³⁴S value below zero.

**
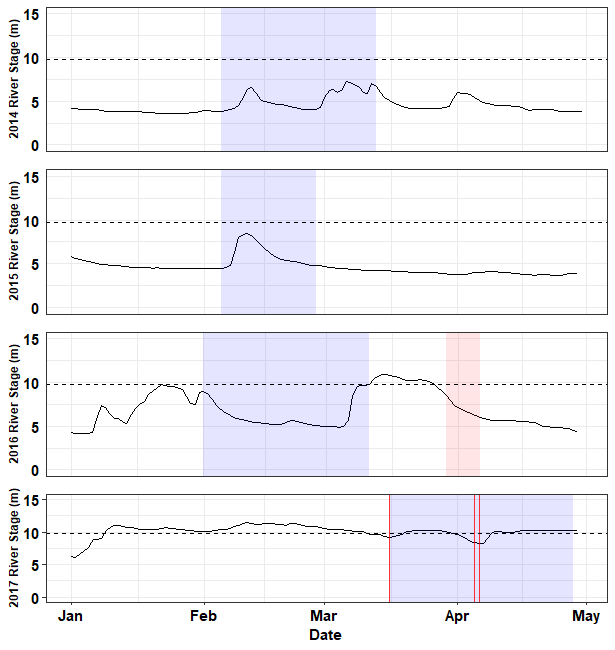
**

## Supplemental Fig S2

Flow conditions on the Yolo Bypass during cage experiments. Plot displays the river stage measured at Fremont Weir during each year the experiments took place. The dashed line represents the Sacramento River stage needed to overtop Fremont Weir to flood the bypass. Blue shading represents the duration of when fish were reared in experimental fields or cages. The red lines and shading display opportunistic sampling efforts on the bypass when conditions allowed.

**
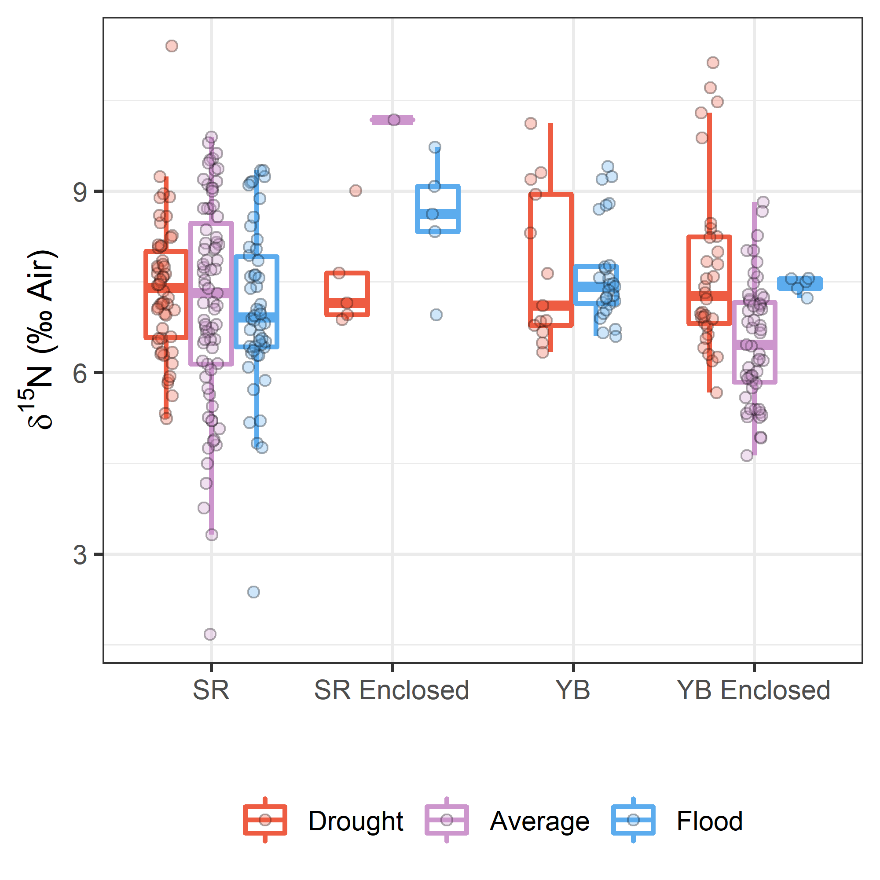
**

## Supplemental Fig S3

δ¹⁵N values of stomach contents from river and floodplain habitats under different hydrologic conditions. The δ¹⁵N values from the stomach contents of fish that reared on the floodplain in the Yolo Bypass were similar to the mean wild-caught and caged river fish throughout all water year types (2012-2015, drought; 2016, average; 1999 and 2017, flood). Box denotes the median and interquartile range, and whiskers denote 95% confidence intervals.

**
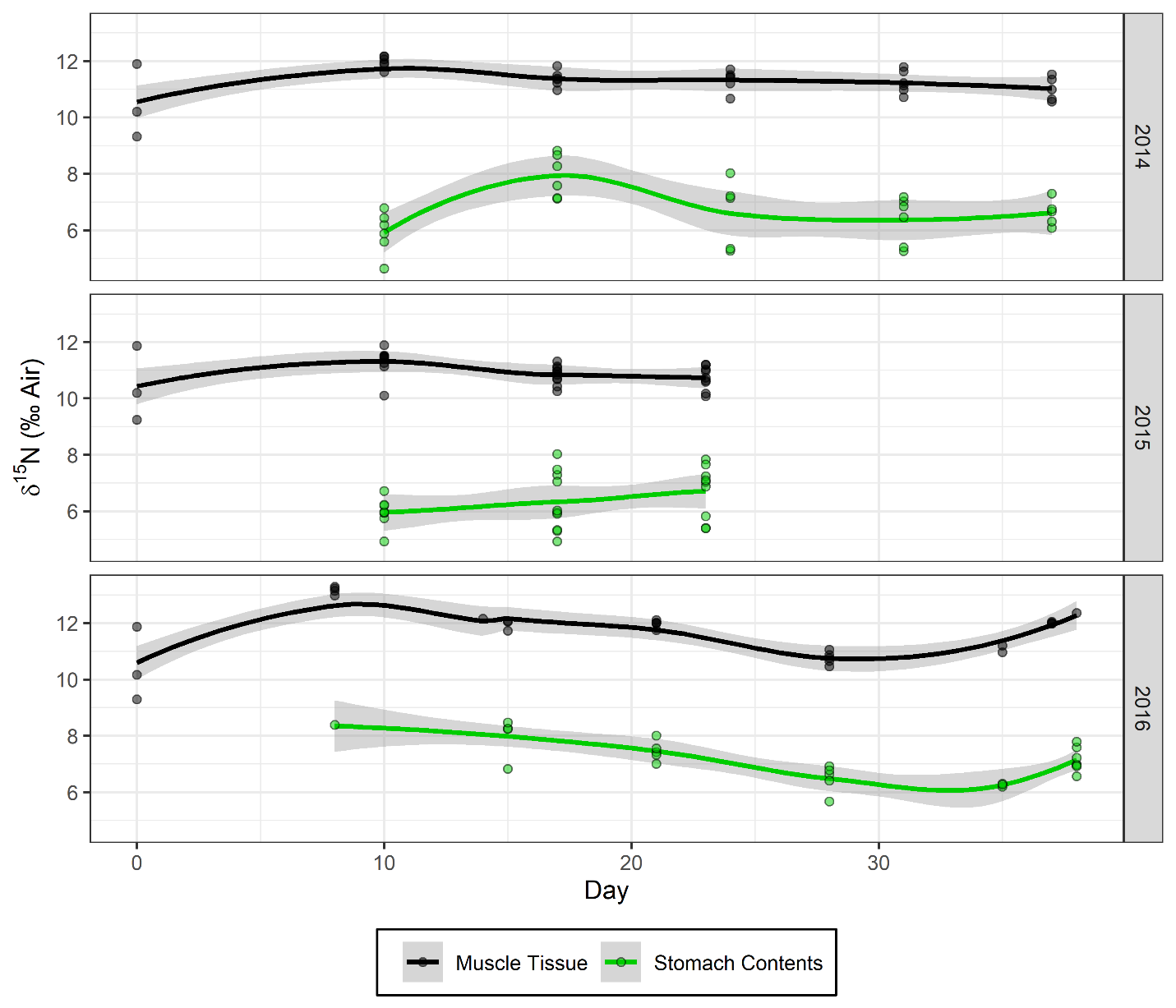
**

## Supplemental Fig S4

Results from δ¹⁵N values tissue assimilation experiment. Each point represents a fish sampled, over the duration of the study. The solid line is a loess smooth (span = 0.75) and shaded regions represent a 95% confidence interval. Fish displayed a ~ 3-4‰ fractionation expected in δ¹⁵N as prey items are integrated into tissue.


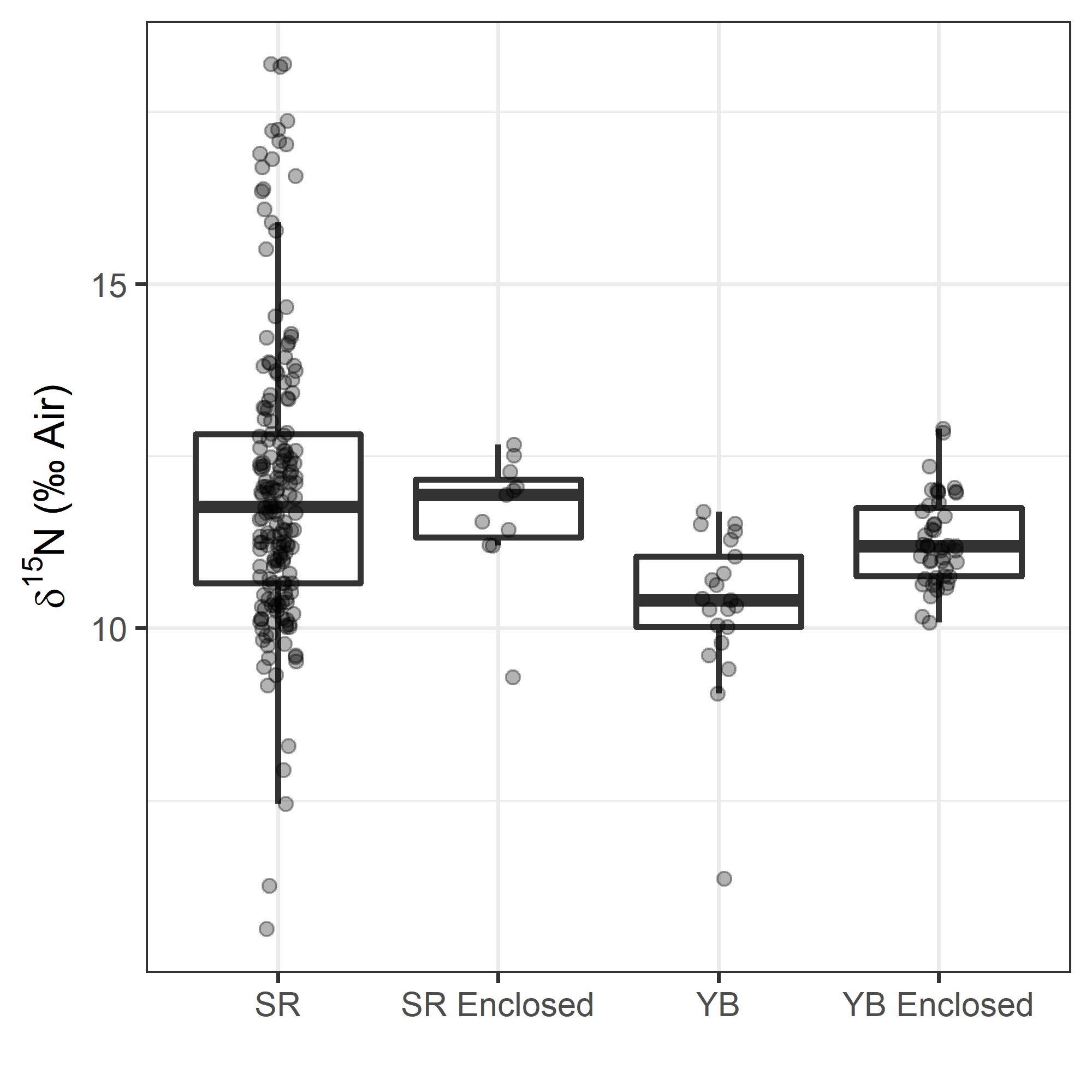


## Supplemental Fig S5

δ¹⁵N values in muscle tissues for wild-caught and enclosed fish in the river and floodplain habitats averaged for all years sampled. Enclosed fish includes caged fish as well as fish held on experimental fields and only muscle tissue from >24 days of the weekly time series were used to show the final isotope values assimilated into tissues.


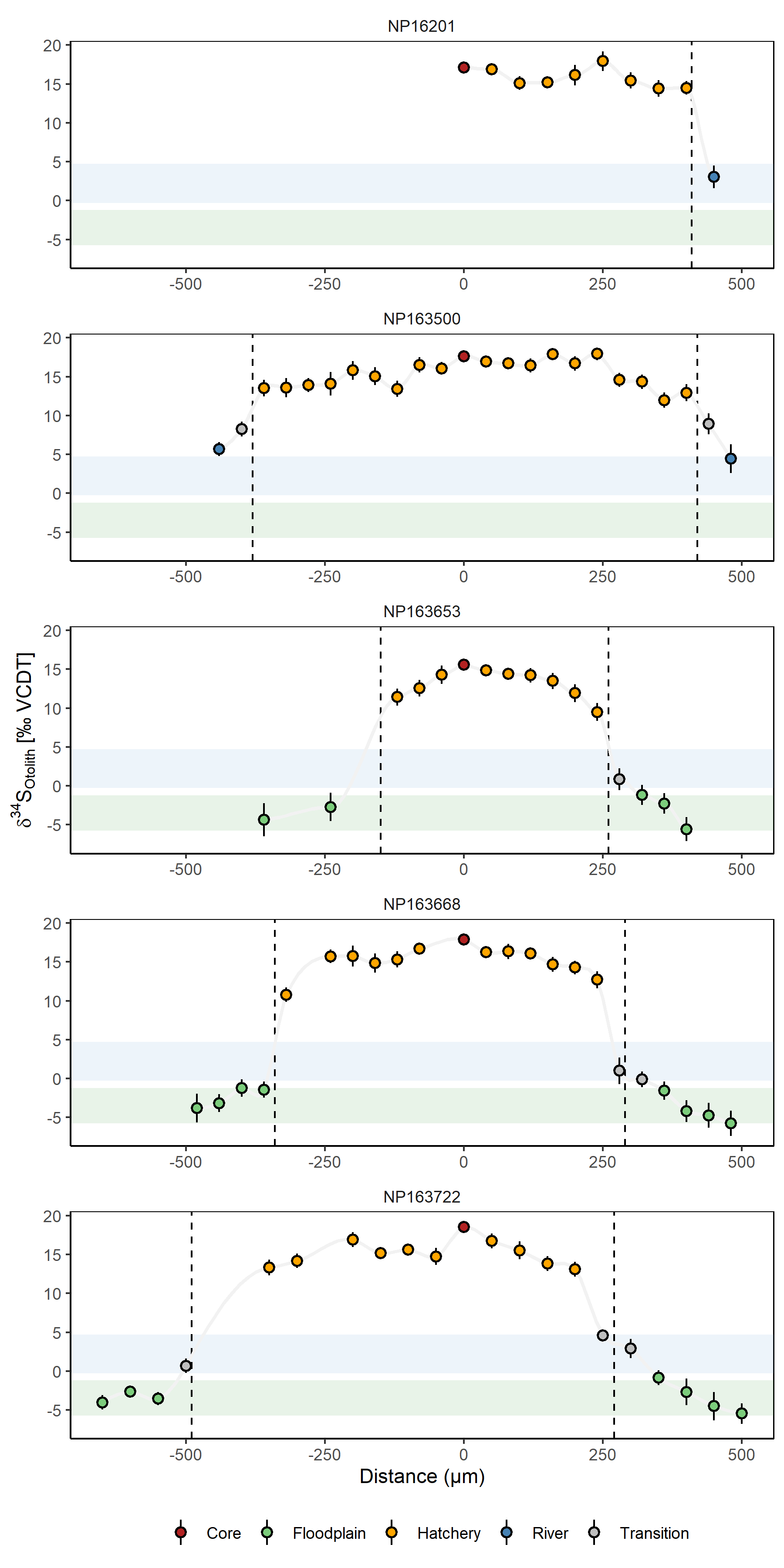


## Supplemental Fig S6

δ³⁴S_Otolith_ profiles for fish reared on the river (n=2) and floodplain (n=3). Spots that overlapped the timing of movement from the hatchery to the river or floodplain were classified as “transition” and not included in the calculation for the δ³⁴S_Otolith_ means as they represent a mixture of otolith growth in different habitats.
