# Supplementary material for: Biogeochemical processes create distinct isotopic fingerprints to track floodplain rearing of juvenile salmon
Source: PLoS One. 2021 Oct 28;16(10):e0257444. doi: 10.1371/journal.pone.0257444 (PMC8553044; doi:10.1371/journal.pone.0257444)
Supplement: Attachment — Submitted filename: PLoS One review.docx [file pone.0257444.s002.docx]

**Review of “Biogeochemical processes create distinct isotopic fingerprints to track floodplain rearing of juvenile salmon”**

The manuscript by Bell-Tilcock et al. describes a study to evaluate the use of multiple stable isotopes (with an emphasis on sulfur), to evaluate their utility for identifying floodplain habitat use by juvenile Chinook Salmon. The authors have assembled a nice data set from a multi-year field effort and have provided data that show promise for the utility of sulfur isotopes to contribute to a better understanding of the role of floodplain rearing to the productivity of Chinook Salmon in this system. That being said, the manuscript suffers from a several major issues that should be addressed in a revision. First, there is a lack of clarity in the sources of data being used in various comparisons that confuses the interpretations of results. Data sources used as the response variable appear to be mostly aggregated but include multiple “rearing types” (experimental floodplain pond, caged fish (floodplain and river), trawl collections). The specific source of data is important because the presence or absence of each data type is dependent on years when floodplain inundation occurs vs. when it is simulated (via the experimental ponds) and researcher decisions. Additionally, the data type to be used in each comparison should be carefully considered to make the most meaningful comparisons relative to the study goal. Second, the authors fail to consider multiple confounding issues in their analysis and interpretations that introduce considerable uncertainty for their assertion about the utility of this technique. These should be considered and described explicitly to keep conclusions within the limits of the data collected. Below I discuss these issues in detail and provide line by line comments as well.

*Lack of clarity in data sources used for analysis*

I can appreciate that the authors draw on multiple fish collections for use in the various comparisons that were made and Table 1 attempts to organize this a bit. However, I found myself constantly flipping back and forth to determine what years, and collection/rearing types were included in the various comparisons. This made it very difficult to assess the relevance of the comparisons or if it was appropriate. For example, lines 266-275 describe a t-test to compare floodplain vs. river diets (isotope ratios, and sulfur specifically) and then on 276-288 a mixed effects model is described that also includes floodplain and river as fixed effects but includes random effects as well. The effect of river or floodplain does not seem to be interpreted in the mixed model results but each is examined separately for a subset of effects. The mixed effects model seems sufficient and the t-test can be excluded unless it uses some alternate combination of data. Lines 374-376 suggest that fish captured on the floodplain (outside of the ponds) were not included in this analysis. Is this true? If so, why were they excluded? Figure 4 suggests that the stomach contents isotope ratios (sulfur in particular) of volitionally migrating fish on the floodplain are more similar to volitionally migrating fish in the river relative to the floodplain pond fish. This seems to have important implications for the goal of the study and suggests the specific composition of the response variable is important to interpreting results. This trend warrants discussion and I would even suggest a model with both isotopes as the response and source (pond, trawl, volitional floodplain, caged river) as fixed effects. This could be done in a MANOVA frame work or multi-response permutation procedures, or ANOSIM. Other frameworks may also be appropriate.

The data described in the manuscript could come from multiple sources including, 1) fish from the experimental ponds (caged and free ranging), 2) volitionally migrating fish on the floodplain, 3) caged fish in the river and 4) fish captured in the river in trawls. Comparisons of river vs. floodplain isotope ratios would seem to be confounded with water year (volitionally migrating fish in the floodplain are only available in wet years) that would in turn affect the data types available in each year. Since these analyses are focused on isotope ratios of stomach contents and muscle, one might predict more variation in volitionally migrating fish that could have fed in a variety of habitats prior to entering the floodplain and their residence time is unknown whereas floodplain fish restricted to the ponds might be expected to have a diet of items available within that pond and residence time is known. Indeed the authors touch on this uncertainty in the discussion. The data from the river for caged vs volitionally migrating fish suggest that rearing/collection type is likely to affect diet as reflected by isotope ratios (Fig 5). I can understand that all these nuances cannot be addressed simultaneously (though some exploration of these different sources should be examined). However, it would be useful to have a table describing the data sources that each response variable is composed of and a statement about weather the authors are assuming that all rearing sources are equivalent. Alternatively, a model could be constructed with rearing/collection type as fixed effects.

*Confounding factors affecting interpretations and applications*

The authors state in the first paragraph of the discussion that they have demonstrated δ^34^S is an ideal floodplain habitat marker. However, I am not convinced that the authors have presented data to support that statement. For example, the otolith portion of the study suggests that sulfur isotopes in the environment are integrated into the otolith. However, to identify floodplain rearing requires fish occupy the floodplain for 39 days (according to figure 7). Fish rearing on the floodplain for less time would fall into the river region and have their natal origin misclassified. For example, the flow data in Fig 2 indicate that the floodplain was inundated for less than 39 days in 2016. Thus, any fish rearing on the floodplain and subsequently captured as adults would have a river signature because they did not rear long enough to acquire the floodplain signature. It would be good to know how many inundation events are over that 39-day threshold to evaluate just how well sulfur may work as a marker of floodplain rearing in otoliths.

There is also a noticeable lack of information on sulfur isotopes in the otoliths of Chinook salmon rearing in natal streams. The authors focus on the Yolo Bypass floodplain and the adjacent Sacramento River. However, juvenile Chinook salmon frequently rear in natal streams until reaching the smolt life stage and then quickly migrate through the study area. The isotopic compositions of these otoliths would be important to make sure they could be differentiated from Yolo or Sacramento rearing fish.

The behavior of natural origin fish also need to be considered when asserting the usefulness of sulfur isotopes. The authors appear to have some data related to this topic that could be explored to provide more confidence in this issue. For example, the experimental ponds appear to have long residence times and may provide optimal conditions for S fractionation. However, in a wet year, large volumes of Sacramento River enter the bypass and residence time may vary considerably. Thus, there would be less opportunity for fractionation. The data in figure four suggest that volitionally migrating fish have more similar S signatures in the two habitat types. A statistical test of this fish of this classification (volitionally migrating) would provide greater confidence that this is a technique that could inform fish not exposed to the experimental (confined) condition. The DFA described on lines 269-275 could potentially accomplish this using muscle tissue for fish that volitionally migrate vs. experimental containment.

Finally, the authors describe significant spatial variation in sulfur isotope ratios within the floodplain. This has the potential to introduce uncertainty into estimates of floodplain rearing in otoliths collected at a later life stage. The northern bypass where the experimental ponds are located may not be representative of the lower Yolo Bypass where estuarine influences are greater. The authors touch on some of these qualifications briefly on lines 495-497 and 519-522. However including statements like lines 511-513 is going a bit far given the data presented. This and the other factors described above should be addressed in some way to qualify the statement that δ^34^S is an ideal floodplain habitat marker. That is not to say the data are not important. They represent and important first step to developing this marker. However, it would be good to propose the logical next steps needed to reduce uncertainty in the method and be more upfront about limitations at this stage.

Line by line comments

Line 37-38 I am not sure why identifying floodplain rearing would be important during extreme drought when floodplain inundation would not be expected.

Lines 38-41 I think the time required to reach the river vs. floodplain zone is important because it can lead to misclassification of rearing location.

Line 44-47 Here and in the introduction, the authors assert that sulfur isotope have been ignored or underutilized in tool in differentiating habitat use. In contrast, this is a tool that has been extensively in other areas (particularly salt marsh habitat) to differentiate habitat use. A more thorough literature review on this topic is warranted.

Line 56-57 There is a rich literature on the role of wetlands as nursery habitats including mangroves, salt march, floodplain wetlands and others. A more thorough literature review on this topic is warranted as well.

Line 59 All floodplains would be off-channel

Line 68-69	The study results suggest that characteristic number 1 is only met when fish rear in the floodplain for ≥6 weeks. Otherwise they would be misclassified. This temporal component should be identified in the discussion when discussing the utility of sulfur isotopes.

Lines 86-87. There are many examples of using SIA to identify freshwater rearing habitats. Just a few would include

[Habitat and resource use by larval and juvenile fishes in an arid‐land river (Rio Grande, New Mexico)](javascript:void(0))

AA Pease, J Justine Davis, MS Edwards, TF Turner

Freshwater Biology 51 (3), 475-486

[Landscape-scale hydrologic characteristics differentiate patterns of carbon flow in large-river food webs](javascript:void(0))

DJ Hoeinghaus, KO Winemiller, AA Agostinho

Ecosystems 10 (6), 1019-1033

[Consistent trophic patterns among fishes in lagoon and channel habitats of a tropical floodplain river: evidence from stable isotopes](javascript:void(0))

KA Roach, KO Winemiller, CA Layman, SC Zeug

Acta Oecologica 35 (4), 513-522

Line 89-98. There are other pathways of carbon flow on floodplains. The diet data presented suggest fish are feeding on pelagic zooplankton. The strength of connectivity between the benthic and pelagic pathways should at least be recognized as a source of uncertainty. The sampling design would have benefited from an attempt to differentiated pathways of carbon assimilation to salmon. This would be an excellent next step for developing this technique.

Line 98. There are many examples of sulfur being used in the food web literature.

Line 137-139 It seems that only caged fish should be used for this comparison since there is uncertainty about the location of food acquisitions for volitional migrants. Or a justification should be provided.

Lines 169-187 It would be important here or somewhere to provide a distribution of floodplain inundation duration for the Yolo Bypass. This seems particularly relevant given the finding that 6 weeks of rearing is needed to be able to identify floodplain rearing in otoliths.

Lines 196-199 This suggests only large magnitude floods are being represented by the experiment.

Lines 209-211 It appears that samples from the river were collected in 2012 and 2013 whereas the pond experiment did not start until 2014. This would result in an unbalanced design in the statistical comparisons of floodplain vs river isotope ratios.

Lines 213-215: The 2016 section of the appendix does not describe fish being contained in the floodplain with the same cages used in the river. This should be clarified here.

Line 267-269: This description is confusing. The tested used all fish from all years but was based on means? Is the response variable the value of individual fish or somehow aggregated into means?

Lines 276-288 A better description of mixed effect model is needed. Were assumptions of this model tested? Analyzing sub-location and water year as random effects does not seem justified when there is so much imbalance in the river and floodplain data given the variation in collection types among years.

Lines 290-297: This is a really nice component of the study and should perhaps have more emphasis.

Lines 298-306: This analysis should be more closely linked with the diet switch study above. It could be described as a way to attempt to confirm the results from the study above. Contracting the caged and volitional migrants in the effort would be particularly interesting.

Line 307-309: Five fish seems like a very small sample size. The study is described in the introduction as an experiment but many of the data seem to be collected in an opportunistic way. Consider changing the description of the study as an experiment.

Line 378. The term < 0 0/00 seems strange because it refers to a concentration which could not be negative. The delta notation can be negative because it is a ratio relative to a standard and 0/00makes sense as the unit of standard deviation, but it doesn’t seem logical for a negative value. I could be wrong.

Lines 376-381. Consider a statistical model that uses both isotopes simultaneously as the response variable.

Line 382-384: Including volitional vs. caged into this analysis would be important.

Figure 5. This figure contains important data for interpretation. First, the sulfur isotope ratio does not appear to equilibrate between stomach contents and muscle tissue for either Yolo or Sacramento-origin fish. A statistical test comparing the sulfur isotope ratio of stomach contents relative to muscle would provide a way to test the result of the diet switch experiment in a more natural setting. If they are not equivalent (particularly in the river) it has important implications for how trophic history may influence the isotopic composition of prey and muscle. Additionally, the plots of the caged fish indicate a narrower trophic range (as represented by isotope ratios) relative to volitionally migrating fish. This suggests caging restricts the food sources available to salmon and may suggest that combining caged and free ranged fish in previous comparisons is not warranted.

Lines 404-407 What about trophic diversity? Thee is evidence for this in the river data.

Lines 444-446 I thought all of these fish were hatchery reared? Were wild fish included here? That would seem to confound the analysis.

Lines 482-485: As described above, the temporal component needs to be discussed since fish rearing on the floodplain less than 6 weeks would be misclassified as river reared fish.

Lines 488-489. This was the result of the specific experiment but the data in fig 5 suggest that fish muscle may rarely reach equilibrium with stomach contents though the reasons are not known.

Line 498-500 This is a great idea, but the authors appear to have those data so why not include a mixing model here and apply to the volitional fish.

Line 511-513: This statement is not supported by the data given the large opportunity for misclassification described above.

Line 519-528 Does it have to be off-channel? It seems a next step to recommend would be a better isotopic mapping of available rearing habitat.

Lines 531-536. Again, there are many isotopic studies of fish using S isotopes.

Line 542: Would floodplain inundation be predicted in extreme drought years?

Line 561-572: The model suggested sub-region was an important source of variation. Doesn’t this have potential implications for a retrospective analysis?

Line 577-580: As described above, it appears that fish are feeding in both benthic and pelagic carbon pathways which could affect the S of fish rearing in the floodplain.

Line 590-598: This is a very favorable interpretation of the data since the potential for misclassification of short term (<40 days) floodplain rearing is high. The study presents interesting data and points to other studies needed to get a tool that could be used to do what is described here. However, I don’t find the data presented supports this interpretation. It would be useful here to describe the future studies needed to refine this technique to where it can be applied to ecological and management questions.
