# Supplementary material for: Biogeochemical processes create distinct isotopic fingerprints to track floodplain rearing of juvenile salmon
Source: PLoS One. 2021 Oct 28;16(10):e0257444. doi: 10.1371/journal.pone.0257444 (PMC8553044; doi:10.1371/journal.pone.0257444)
Supplement: Attachment — Submitted filename: Response_to_plos_one_comments-2020-12-18.docx [file pone.0257444.s003.docx]

*Reviewer 1*

*L92: For describing isotope values, authors should consider using high/low instead of enriched/depleted because d13C are numbers and they cannot be depleted or enriched. This expression should be reflected throughout the manuscript.*

Response: Thank you for the suggestion, this has now been fixed throughout the MS*.*

*L366: Fig. “3” instead of Fig. “2”*

Response: This is now Fig 2 since the original figure 2 was removed. Line 416

*L387: Fig. “3” instead of Fig. “2”. Please also indicate what the ellipses in the figure represent*

Response: See comment above ; Ellipses represent 95% CI and now included in the figure legend. Line 440

*L420: Please indicate the alpha value used for statistical significance in the methods because water year type (P = 0.019) could become significant with an alpha value of 0.05*

Response: The mixed effects model has been revised per reviewer 2’s suggestion of only using years that have a direct river to floodplain comparison. Some of the years were removed from the model and all significance levels are compared to traditional alpha 0.05. Line 296; 321-322

*L431: Fig. “6” instead of Fig. “5”*

Response: This is now Fig 5 since Fig 2 was removed. Line 492;493;496

*L442: Please indicate what the solid lines represents in the figure.*

Response: Solid line has been indicated as a loess smooth with span = 0.75 in figure legend. *Line 513*

*Reviewer 2*

*The claim that sulfur met all the authors criteria as a floodplain marker and can be used in a retrospective analysis of floodplain use is not supported by the data. The data clearly indicate that fish rearing less than 39 days on the floodplain would be classified as river-reared fish. This means that the ability of S to differentiate habitats is dependent on rearing time which would hamstring a retrospective analysis for years when inundation is less than 39 days.*

*Response: We have altered the text in the introduction to include equilibration rates as an important component of an ideal floodplain marker (Line 102-105). Additionally, we have added additional text in the discussion demonstrating how this study can be used to identify floodplain rearing of less than 39 days using methods detailed in Moore et al., 2016. Lastly, the otolith data used in this paper has multiple spots read on the otolith, each representing ~10-15 days with floodplain rearing values similar to what was found in the stomachs and muscle. Finer temporal resolution is possible using the otoliths. This has been added to the discussion. Line 584-623*

*The statistical analysis treats all fish samples as equivalent however, their "rearing type" is variable (caged, experimental pond, volitional migrating). Figures 4 and 5 suggest that rearing type influences isotopic composition. Since the data are unbalanced in regards to fixed effects, an analysis where these confounding influences are accounted for is warranted. Alternatively, analyses could be performed with subsets of the data that are more comparable.*

*Response: The mixed effects models in this study did include “rearing type” as a variable and fish were not treated as equivalent. This is now explained more clearly in the methods to specifically state what data our model includes and the structure of the model. Line 297-324*

*First, there is a lack of clarity in the sources of data being used in various comparisons that confuses the interpretations of results. Data sources used as the response variable appear to be mostly aggregated but include multiple “rearing types” (experimental floodplain pond, caged fish (floodplain and river), trawl collections). The specific source of data is important because the presence or absence of each data type is dependent on years when floodplain inundation occurs vs. when it is simulated (via the experimental ponds) and researcher decisions. Additionally, the data type to be used in each comparison should be carefully considered to make the most meaningful comparisons relative to the study goal. Second, the authors fail to consider multiple confounding issues in their analysis and interpretations that introduce considerable uncertainty for their assertion about the utility of this technique. These should be considered and described explicitly to keep conclusions within the limits of the data collected.*

*Response: We appreciate the concerns and attention to our analysis. We agree the fish do need to be treated differently depending on rearing type, which our model did capture. This was explained under the variable “subsite” in the R code, which included the locations of where the fish were captured, ie. experimental ponds, seined in different locations, captured in the rotary screw trap. We agree that this was not clearly articulated in the methods of the manuscript and have adjusted the wording to further explain this. We have also added separate models to address the δ¹³C variation as well, since this was not originally included. Line 297-324*

*Additionally, in Figure 3 (formally Figure 4), we highlight which fish were volitional vs those reared in ponds or cages to also demonstrate how even the volitional fish on the floodplain were similar to the pond reared counterparts.*

*For example, lines 266-275 describe a t-test to compare floodplain vs. river diets (isotope ratios, and sulfur specifically) and then on 276-288 a mixed effects model is described that also includes floodplain and river as fixed effects but includes random effects as well. The effect of river or floodplain does not seem to be interpreted in the mixed model results but each is examined separately for a subset of effects. The mixed effects model seems sufficient and the t-test can be excluded unless it uses some alternate combination of data.*

*Response: T-test has been removed for each isotope with the exception of δ¹⁵N, since this isotope showed the lowest variation and was not used as a floodplain marker or in the models. Line 471-476*

*Lines 374-376 suggest that fish captured on the floodplain (outside of the ponds) were not included in this analysis. Is this true? If so, why were they excluded?*

*Response: The fish captured outside of the ponds were included in this but we agree that the original text makes it appear otherwise. This has now been fixed. Line 297-324*

*Figure 4 suggests that the stomach contents isotope ratios (sulfur in particular) of volitionally migrating fish on the floodplain are more similar to volitionally migrating fish in the river relative to the floodplain pond fish. This seems to have important implications for the goal of the study and suggests the specific composition of the response variable is important to interpreting results. This trend warrants discussion and I would even suggest a model with both isotopes as the response and source (pond, trawl, volitional floodplain, caged river) as fixed effects. This could be done in a MANOVA frame work or multi-response permutation procedures, or ANOSIM. Other frameworks may also be appropriate.*

*Response: While there is overlap in the sulfur values found in the invertebrate prey items, it is more likely due to river fish previously feeding on either subsidies from off-channel habitats or rearing in another floodplain. Despite the potential for fish having “moved” between habitats prior to capture, the LDA still predicted habitat of capture with high accuracy and uses both δ¹³C and δ³⁴S simultaneously for this prediction. More has been added in the discussion about this point. Line: 623-653*

*The data described in the manuscript could come from multiple sources including, 1) fish from the experimental ponds (caged and free ranging), 2) volitionally migrating fish on the floodplain, 3) caged fish in the river and 4) fish captured in the river in trawls. Comparisons of river vs. floodplain isotope ratios would seem to be confounded with water year (volitionally migrating fish in the floodplain are only available in wet years) that would in turn affect the data types available in each year. Since these analyses are focused on isotope ratios of stomach contents and muscle, one might predict more variation in volitionally migrating fish that could have fed in a variety of habitats prior to entering the floodplain and their residence time is unknown whereas floodplain fish restricted to the ponds might be expected to have a diet of items available within that pond and residence time is known. Indeed the authors touch on this uncertainty in the discussion. The data from the river for caged vs volitionally migrating fish suggest that rearing/collection type is likely to affect diet as reflected by isotope ratios (Fig 5). I can understand that all these nuances cannot be addressed simultaneously (though some exploration of these different sources should be examined). However, it would be useful to have a table describing the data sources that each response variable is composed of and a statement about weather the authors are assuming that all rearing sources are equivalent. Alternatively, a model could be constructed with rearing/collection type as fixed effects.*

*Response: As addressed in response to the previous comment, above, the model accounts for variation due to ‘subsite’ which differentiates the volitional from experimental pond or caged fish. This has now been clarified and added to the paper along with a model that also looks at the variation in δ¹³C values as well. In the model, we found that water year type did not have significant effect on the stomach content sulfur value but it did have an effect on muscle tissue, which is likely due to some juvenile fish using the bypass as a migratory corridor instead of rearing habitat. The model has been explained more thoroughly in the methods (Line = 296-323) and we have added more to the discussion detailing how these data could be used to estimate floodplain rearing in fish exposed to the floodplain over a range of durations (Line 584-622).*

*The authors state in the first paragraph of the discussion that they have demonstrated δ^34^S is an ideal floodplain habitat marker. However, I am not convinced that the authors have presented data to support that statement. For example, the otolith portion of the study suggests that sulfur isotopes in the environment are integrated into the otolith. However, to identify floodplain rearing requires fish occupy the floodplain for 39 days (according to figure 7). Fish rearing on the floodplain for less time would fall into the river region and have their natal origin misclassified. For example, the flow data in Fig 2 indicate that the floodplain was inundated for less than 39 days in 2016. Thus, any fish rearing on the floodplain and subsequently captured as adults would have a river signature because they did not rear long enough to acquire the floodplain signature. It would be good to know how many inundation events are over that 39-day threshold to evaluate just how well sulfur may work as a marker of floodplain rearing in otoliths.*

*Response:* The reviewer is referring to the definition of a floodplain rearing marker *as “*An ideal floodplain rearing marker would 1) be distinguishable from other possible salmon rearing habitats across the landscape 2) remain stable within season and among years, and 3) become integrated into tissues at known equilibration rates as well as permanently stored in archival tissue”. The reviewer is correct; it can take some time for the dietary value to become integrated but the time component is only related to what tissue is being analyzed as different tissues have different integration rates. However, our otolith data shows multiple spots recorded with the floodplain marker. These spots represent ~10-15 days but higher spatial resolution is achievable when measuring δ³⁴S in an otolith to be less than a week. So while the muscle tissue might not equilibrate, the otolith will permanently archive the floodplain marker which does demonstrate this is an ideal floodplain marker. This has been addressed in the discussion. Line: 584-622

*There is also a noticeable lack of information on sulfur isotopes in the otoliths of Chinook salmon rearing in natal streams. The authors focus on the Yolo Bypass floodplain and the adjacent Sacramento River. However, juvenile Chinook salmon frequently rear in natal streams until reaching the smolt life stage and then quickly migrate through the study area. The isotopic compositions of these otoliths would be important to make sure they could be differentiated from Yolo or Sacramento rearing fish.*

*Response:* For the otoliths used in this study, we knew the natal origins of the salmon as they all came from the same hatchery and were placed into the habitats of interest. As for the application to wild fish, we agree more work to describe the sulfur isoscape in the Central Valley of California needs to be done. In the discussion, we do identify the lack of the data available using sulfur in this region and discuss how multiple isotope systems need to be used to reconstruct a more comprehensive life history for juvenile salmon. Line: 632-653

*The behavior of natural origin fish also need to be considered when asserting the usefulness of sulfur isotopes. The authors appear to have some data related to this topic that could be explored to provide more confidence in this issue. For example, the experimental ponds appear to have long residence times and may provide optimal conditions for S fractionation. However, in a wet year, large volumes of Sacramento River enter the bypass and residence time may vary considerably. Thus, there would be less opportunity for fractionation. The data in figure four suggest that volitionally migrating fish have more similar S signatures in the two habitat types. A statistical test of this fish of this classification (volitionally migrating) would provide greater confidence that this is a technique that could inform fish not exposed to the experimental (confined) condition. The DFA described on lines 269-275 could potentially accomplish this using muscle tissue for fish that volitionally migrate vs. experimental containment.*

*Response:* We agree that there is variability in higher water years and we do include this in our data analysis as well as discuss it further in the discussion. Briefly, many of the natural origin fish captured in 2017, California’s wettest year on record, still exhibited sulfur values less than 0 in both stomach contents and muscle tissues. These values, while not as reduced as in the previous years, were still negative and still more reduced compared to those in the river. Demonstrating that the same biogeochemical processes occur, even when there are large amounts of water. This was also tested in the mixed effects model with water year type as a random effect. Additionally, Figure 4 has been altered to display these water year differences more clearly.

Volitional fish in this study from 2016 and 2017 were had values in their stomach contents and muscle tissues that were similar to those of the caged fish from previous years. This can be seen in the LDA plot (Figure 3) with the open circle fish still having more reduced values compared to the river fish and these fish were accurately classified as floodplain fish.

*Finally, the authors describe significant spatial variation in sulfur isotope ratios within the floodplain. This has the potential to introduce uncertainty into estimates of floodplain rearing in otoliths collected at a later life stage. The northern bypass where the experimental ponds are located may not be representative of the lower Yolo Bypass where estuarine influences are greater. The authors touch on some of these qualifications briefly on lines 495-497 and 519-522. However including statements like lines 511-513 is going a bit far given the data presented. This and the other factors described above should be addressed in some way to qualify the statement that δ^34^S is an ideal floodplain habitat marker. That is not to say the data are not important. They represent and important first step to developing this marker. However, it would be good to propose the logical next steps needed to reduce uncertainty in the method and be more upfront about limitations at this stage.*

*Response:* While this did cause the largest variation in δ³⁴S isotope values found in the fish captured on the bypass, these values were typically less than 0. At this point, no other rearing habitat has been identified to have δ³⁴S values less than 0, and a more exhaustive search on the landscape needs to be done for future work. Additionally, these fish were still accurately classified as floodplain fish, even with the variation, demonstrating that this should not have impact on retroactive analysis.

*Line 37-38 I am not sure why identifying floodplain rearing would be important during extreme drought when floodplain inundation would not be expected.*

Response: While the Yolo Bypass does not flood every year, the Sutter Bypass does flood almost every year including most drought years. The main point is that we had an opportunity to test the extent to which the floodplain markers were robust spatial markers over a range of hydrological conditions, which we tested and confirmed and may be particularly relevant to other study systems. This has been clarified in the discussion (Line 638-651).

*Lines 38-41 I think the time required to reach the river vs. floodplain zone is important because it can lead to misclassification of rearing location.*

*Response: A time component has been added into the abstract (weekly for otoliths). Additionally, see above comments about rearing time. Line 38-39*

*Line 44-47 Here and in the introduction, the authors assert that sulfur isotope have been ignored or underutilized in tool in differentiating habitat use. In contrast, this is a tool that has been extensively in other areas (particularly salt marsh habitat) to differentiate habitat use. A more thorough literature review on this topic is warranted.*

*Response:* Sulfur isotopes compared to CN have largely been underutilized in aquatic ecology. However, we have included more references, particularly those in salt marsh habitats that have used S isotopes to differentiate habitat use.

*Line 56-57 There is a rich literature on the role of wetlands as nursery habitats including mangroves, salt march, floodplain wetlands and others. A more thorough literature review on this topic is warranted as well*

*Response:* We agree, there have been many studies that have looked as these wetland habitats as nursery function, here we are specifically using the Beck et al definition identifying habitats that disproportionally contribute to the survival of individuals. Identifying where adult individuals reared has been lacking. This wording has been clarified and citations have been added. Line 123-124

*Line 59 All floodplains would be off-channel*

*Response: “off-channel” has been removed. Line 91*

*Line 68-69	The study results suggest that characteristic number 1 is only met when fish rear in the floodplain for ≥6 weeks. Otherwise they would be misclassified. This temporal component should be identified in the discussion when discussing the utility of sulfur isotopes.*

*Response: The first criteria of an ideal floodplain marker is* 1) be distinguishable from other possible salmon rearing habitats across the landscape. The invertebrate stomach content data suggests that the two habitat types are distinct from one another using sulfur and carbon isotopes. Our data does indeed support the first criteria and does not need to include a temporal component for it to fulfilled. The reviewer may be referencing the equilibrium rate into muscle tissue as having a time component. We have added language discussing the different resolution of tissues and the value in archival tissues [like otoliths] that can record diet shifts on the daily to weekly interval (Line 610-622). See also our previous response to this issue.

*Lines 86-87. There are many examples of using SIA to identify freshwater rearing habitats. Just a few would include*

[*Habitat and resource use by larval and juvenile fishes in an arid‐land river (Rio Grande, New Mexico)*](javascript:void(0))

*AA Pease, J Justine Davis, MS Edwards, TF Turner*

*Freshwater Biology 51 (3), 475-486*

[*Landscape-scale hydrologic characteristics differentiate patterns of carbon flow in large-river food webs*](javascript:void(0))

*DJ Hoeinghaus, KO Winemiller, AA Agostinho*

*Ecosystems 10 (6), 1019-1033*

[*Consistent trophic patterns among fishes in lagoon and channel habitats of a tropical floodplain river: evidence from stable isotopes*](javascript:void(0))

*KA Roach, KO Winemiller, CA Layman, SC Zeug*

*Acta Oecologica 35 (4), 513-522*

*Response: Sentence has been modified and additional citations have been added. Line 123-124*

*Line 89-98. There are other pathways of carbon flow on floodplains. The diet data presented suggest fish are feeding on pelagic zooplankton. The strength of connectivity between the benthic and pelagic pathways should at least be recognized as a source of uncertainty. The sampling design would have benefited from an attempt to differentiated pathways of carbon assimilation to salmon. This would be an excellent next step for developing this technique.*

Response: We agree, this would be a great next step for better understanding the isotopic food web on floodplains and how it can be used as a floodplain marker and has been added in the discussion. One study done in the Yolo Bypass has found carbon to be sourced from heterotrophic pathways and has been added in the discussion (line). There is also an ongoing experiment with collaborators at UC Davis, addressing this question in more detail. Additional information with citations have been added. Line 704-705

*Line 98. There are many examples of sulfur being used in the food web literature.*

*Response: This sentence has been removed.*

*Line 137-139 It seems that only caged fish should be used for this comparison since there is uncertainty about the location of food acquisitions for volitional migrants. Or a justification should be provided.*

Response: The volitional floodplain fish were all captured either on the floodplain or as they were exiting the floodplain and were presumably feeding locally. This is why they were included along with the caged fish*.*

*Lines 169-187 It would be important here or somewhere to provide a distribution of floodplain inundation duration for the Yolo Bypass. This seems particularly relevant given the finding that 6 weeks of rearing is needed to be able to identify floodplain rearing in otoliths.*

Response: Appendix Figure B shows the inundation period of the Yolo Bypass in various water years. In general, inundation occurs over multiple weeks. Additionally, our data found values go below zero beginning around 4 weeks but most of our experiments took place over the course of 6 weeks. In 2016 for example, natural inundation occurred beginning March 12 and then disconnection occurred April 8^th^. During this time, we had no experimental pond fish due to the incoming high flows and fish were released. We did capture fish that were leaving the bypass, and the fish that had stayed and reared on the bypass at that time had values in their tissues and stomach contents similar to those of the experimental ponds which were δ³⁴S less than zero and lower than any river value. Lastly, it would not take 6 weeks for this habitat marker to be archived in the otolith. Our study had 3 spots measured in the otolith, each representing ~10-15 days spent on the floodplain. It is also possible to look at δ³⁴S on a finer temporal scale than our study did.

*Lines 196-199 This suggests only large magnitude floods are being represented by the experiment.*

Response: The word “large” has been removed since they year 2016 represents an average water year with a smaller flood event. Line 243

*Lines 209-211 It appears that samples from the river were collected in 2012 and 2013 whereas the pond experiment did not start until 2014. This would result in an unbalanced design in the statistical comparisons of floodplain vs river isotope ratios.*

Response: Good suggestion, the years above have been removed from the model as well as the year 1999 since there is not a direct river comparison for that year as well. These years were part of the pilot data, and since they only contain δ³⁴S values with none of the other isotopes, they were not originally included in the LDA. The models have been revised to only include years with both floodplain and river values. However, data for all years are provided in the appendix. Line 296-323

*Lines 213-215: The 2016 section of the appendix does not describe fish being contained in the floodplain with the same cages used in the river. This should be clarified here.*

Response: This has been fixed with the correct cage specifications. Line 207

*Line 267-269: This description is confusing. The tested used all fish from all years but was based on means? Is the response variable the value of individual fish or somehow aggregated into means?*

Response: The t-test has been removed and model results reported per previous suggestion. However, the t-test was kept for the δ¹⁵N comparison since these values were not included in any of the models due to their similarity between the two habitats.

*Lines 276-288 A better description of mixed effect model is needed. Were assumptions of this model tested? Analyzing sub-location and water year as random effects does not seem justified when there is so much imbalance in the river and floodplain data given the variation in collection types among years.*

Response: The model assumes water year type and sub-location as random effects because of the high variability of the type of rain events California experiences each year. Water year type groups the different years together into three main categories we are likely to see in California (drought, average, and wet). This accounts for the difference in sampling between the years so that they are more balanced. The sub –location or where a fish was captured was included in the model because as there is more water available on the landscape, this provides more sampling opportunities, particularly on the floodplain. Additional text has been added for clarification and only years with river and floodplain data were used in the model. Line 296-323

*Lines 290-297: This is a really nice component of the study and should perhaps have more emphasis.*

Response: Agreed. Additional text has been added in the discussion. Line 583-595

*Lines 298-306: This analysis should be more closely linked with the diet switch study above. It could be described as a way to attempt to confirm the results from the study above. Contracting the caged and volitional migrants in the effort would be particularly interesting.*

*Response:* Thank you for the suggestion, this is currently outside of the scope of the study and the focus of future efforts. Our study aims to provide the foundation to use the depuration curves created in this manuscript to better understand the volitional fish in future studies.

*Line 307-309: Five fish seems like a very small sample size. The study is described in the introduction as an experiment but many of the data seem to be collected in an opportunistic way. Consider changing the description of the study as an experiment.*

Response: We provided these analyses from focal comparisons as a proof-of-concept for future ecological applications.

*Line 378. The term < 0 0/00 seems strange because it refers to a concentration which could not be negative. The delta notation can be negative because it is a ratio relative to a standard and 0/00makes sense as the unit of standard deviation, but it doesn’t seem logical for a negative value. I could be wrong.*

Response: This was part of the t-test results, which has been removed.

*Lines 376-381. Consider a statistical model that uses both isotopes simultaneously as the response variable.*

Response: The LDA uses both isotopes simultaneously to assign habitat-of-origin, additionally, mixed effect models looking at δ¹³C values in stomach contents has been added to the text. Line 444-454

*Line 382-384: Including volitional vs. caged into this analysis would be important.*

Response: Caged vs volitional fish are highlighted in Figure 3 (formally Figure 4; Line 439) with either open circles denoting naturally recruited fish or closed circles for caged/experimental pond fish. They were also used in the assignment, which still yielded high accuracy with river vs floodplain fish.

*Figure 5. This figure contains important data for interpretation. First, the sulfur isotope ratio does not appear to equilibrate between stomach contents and muscle tissue for either Yolo or Sacramento-origin fish. A statistical test comparing the sulfur isotope ratio of stomach contents relative to muscle would provide a way to test the result of the diet switch experiment in a more natural setting. If they are not equivalent (particularly in the river) it has important implications for how trophic history may influence the isotopic composition of prey and muscle. Additionally, the plots of the caged fish indicate a narrower trophic range (as represented by isotope ratios) relative to volitionally migrating fish. This suggests caging restricts the food sources available to salmon and may suggest that combining caged and free ranged fish in previous comparisons is not warranted.*

Response: Figure 4 (formally Figure 5) was not intended to show equilibration in the sulfur values, as it was meant to show the range of values experienced in either habitat. As mentioned in the description of the figure, the variation in muscle tissue was due to the fish starting from the hatchery and reaching equilibrium over time. However, this plot has been changed to just display the stomach contents by water year type since the stomach content values represent the day of capture whereas the muscle tissue is averaged throughout the experiment. Figure 5 (formally fig 6) is where we show the equilibration of tissues over time. This is a better representation of equilibrium in tissues because it shows fish rearing at multiple time intervals and directly compares an individual’s muscle tissue with the same individual’s stomach contents.

*Lines 404-407 What about trophic diversity? Thee is evidence for this in the river data.*

Response: Throughout most of the years on the floodplain, there was very little trophic diversity in the stomach contents. While this is not detailed in this manuscript, there is a more detailed manuscript currently in review but available for preprint (Holmes et al., 2020; Line 683) that discusses this more thoroughly. It is currently outside the scope of this specific study but this is now cited in this manuscript.

*Lines 444-446 I thought all of these fish were hatchery reared? Were wild fish included here? That would seem to confound the analysis.*

Response: Yes, these were all hatchery reared fish and the wording has been changed for clarification. Line 536

*Lines 482-485: As described above, the temporal component needs to be discussed since fish rearing on the floodplain less than 6 weeks would be misclassified as river reared fish.*

Response: See responses above for detailed explanation.

*Lines 488-489. This was the result of the specific experiment but the data in fig 5 suggest that fish muscle may rarely reach equilibrium with stomach contents though the reasons are not known.*

Response: See comment above regarding Figure 5 (now Fig 4). This was not intended to show equilibration since this plot represents fish captured in both habitats at different points in time. Figure 5 (formally Fig 6) is where there is data showing fish rearing in one location for a set period of time to better understand equilibration.

*Line 498-500 This is a great idea, but the authors appear to have those data so why not include a mixing model here and apply to the volitional fish.*

Response: We agree but this is getting outside the scope of our study which is creating the foundation for this type of work.

*Line 511-513: This statement is not supported by the data given the large opportunity for misclassification described above.*

Response: See detailed response above under major revisions regarding misclassification.

*Line 519-528 Does it have to be off-channel? It seems a next step to recommend would be a better isotopic mapping of available rearing habitat.*

Response: We agree, there needs to be greater isotopic mapping of available rearing habitat, which we suggest as future work. We are currently assuming off-channel, since the values are similar and the way those isotopic values become reduced are from habitats with these biogeochemical characteristics. Line 714-721

*Lines 531-536. Again, there are many isotopic studies of fish using S isotopes.*

*Response: This has been removed.*

*Line 542: Would floodplain inundation be predicted in extreme drought years?*

*Response: See response above at “Line 37-38” for detailed answer on this topic.*

*Line 561-572: The model suggested sub-region was an important source of variation. Doesn’t this have potential implications for a retrospective analysis?*

Response: While this did cause the largest variation in δ³⁴S isotope values found in the fish captured on the bypass, these values were typically less than 0. At this point, no other rearing habitat has been identified to have δ³⁴S values less than 0, and a more exhaustive search on the landscape needs to be done for future work. Additionally, these fish were still accurately classified as floodplain fish, even with the variation, demonstrating that this should not have impact on retroactive analysis.

*Line 577-580: As described above, it appears that fish are feeding in both benthic and pelagic carbon pathways which could affect the S of fish rearing in the floodplain.*

*Response: See response to comment “Line 89-98”, this is an interesting point but is currently outside the scope of the current study. While this study does not address the specific fractionation pathways, this does not influence the utility of C and S as floodplain markers.*

*Line 590-598: This is a very favorable interpretation of the data since the potential for misclassification of short term (<40 days) floodplain rearing is high. The study presents interesting data and points to other studies needed to get a tool that could be used to do what is described here. However, I don’t find the data presented supports this interpretation. It would be useful here to describe the future studies needed to refine this technique to where it can be applied to ecological and management questions.*

*Response: More text has been added in the discussion to demonstrate the utility of this study and how it could be used to identify floodplain rearing/residence in future work. We have bolstered the language describing our data on how otoliths record the sulfur habitat signature on the order of days. This language as well as language about how isotope mixing models can assign duration of floodplain rearing in fish that have spent <40 days on the floodplain aims to better explain the utility of this work to address ecological and management questions. Line 610-652*

*Reviewer 3*

*Introduction would be strengthened by doing some re-organizing. First paragraph is great, starts with the broad, global importance of wetlands, but then jumps into salmon without much of a link. I would suggest splitting the first paragraph at line 59, and then adding some detail onto the salmon part.

The rest of the introduction also seems to go back and forth between specific applications of SIA to salmon in California, and broader development of methods and approaches to understand migratory history of animals. I think a bit of restructuring could help to go from the broadest ideas (i.e. wetlands are important, but hard to track importance) to narrower (i.e. SIA can help) to narrowest (i.e. SIA can help understand dynamics of California Chinook).*

Response: Thank you for the suggestion, we agree and have reorganized the introduction to improve readability.

*L266-267: From the text here, it’s not super clear how stomach content differences were tested/modelled? Were all stomachs pooled from floodplain and river? Across all years? Sites? Fig 1 looks like everything was pooled, but L548-550 make it sound like annual comparisons were made*

*Response: All stomachs were pooled together for all years in the floodplain and the river. The text has been modified to highlight the diet studies from this same study area in the different years to highlight the taxonomic differences. Additional text has been added to improve clarity on which specific data is being modeled. Line 296-323*

*L494-495: The Woodson reference is interesting because it appears that size is most important in years of low recruitment, perhaps this is worth highlighting here (especially since it looks like ocean conditions are not getting more favourable for California Chinook (e.g. Wells et al. 2016))

Wells, B. K., Santora, J. A., Schroeder, I. D., Mantua, N., Sydeman, W. J., Huff, D. D., & Field, J. C. (2016). Marine ecosystem perspectives on Chinook salmon recruitment: a synthesis of empirical and modeling studies from a California upwelling system. Marine Ecology Progress Series, 552, 271-284.*

Response: Good suggestion, this has now been added into the discussion. Line 580-582

*L 98: Maybe reword to “d34S is less often used in the isotope ecology literature”*

Response: This has been removed.

*L150 remove “during their early ocean years”*

Response: This has been removed.

*L234 – Fig 2 could go in supplement*

Response: This has now been moved to the Supplemental and is now Supplemental B. Line 1174

*L 302 – list the random effects here again*

Response: The original description of the model has been altered. Line 296-323. Per Reviewer 2’s suggestion, only the stomach contents have been modeled and the muscle tissue has not been subjected to statistical analysis due to the variability in turnover times and unknown turnover rates. The muscle tissues are still described in the results section. Line 518-534

*L302 – might be worth including a sentence on why you thought these random effects might influence assimilation rates of d34S.*

Response: Text has been added to clarify why each random effect was selected. Line 296-323

*L418-420 – if this is still not statistically significant, is it worth pointing out?*

Response: Water year type is so variable in California and is a question asked very frequently. It is important that water year type had little to no effect on the dietary isotope values found on the floodplain.

*L475 – 487 – I would remove the references from Oele et al from this paragraph and put them later on. Good material, but I think this paragraph is stronger if you just summarize your own work*

Response: Thank you for the suggestion, this has been removed.

*L511-513: I would add a “potentially” here. This is likely true, but this paper doesn’t use adult otoliths, so this is speculative*

Response: This has been added.

*L585-598: Remove references to eye lenses here, not covered in this paper*

Response: The references were left in here but altered the wording to say that eye lenses representing a potential permanent archive for diet reconstruction. Since lenses have been shown to accurately record diet based isotopes, they are another option we could use in the future.

*Reviewer #4: This ms might be better off as several separate ms enabling greater discussion of each of the various approaches. Would have liked to see the C-13 & N-15 data for the amphipods to match the fig in app A. IMHO the most problematic aspect in using S-34 is that there are 3 endmembers - one the very enriched marine value, two the slightly negative reduced value seen in Yolo and three the slightly positive "normal" freshwater endmember. This latter one seems to vary by location with First Mallard and Lindsey being the most positive. Why was this so? Yolo is not quite as negative in S-34 in the wet year - is this evidence of a nutrient subsidy from the main river? A bit disappointed in only N=5 otoliths in two categories so only N=2 and N=3 per group!!!*

Response: Thank you for the suggestion, however we disagree and believe the data needs to be presented together to tell a complete story.

The amphipods and the isoscape was put in the appendix due to there not being replicates of the samples and only sampled in one of the years. The variation seen in the amphipods is due to sulfur values following a salinity gradient, becoming higher with increasing salinity. This is the enrichment you are seeing in the isoscape, since those areas are not as fresh as the sites above as noted in the manuscript as well. (line 123-124).

As for the 3 endmembers, this was the variation we were wanting to study so that we could determine the salmon rearing habitats. This large variation is particularly useful to study salmon in the Central Valley.

As for the less negative values in a wet year, this is due to the increased amount of water diluting the signal. However, it’s important to note that the value was still negative and lower than the river, allowing for the correct classification between the habitats.

Sulfur in-situ analysis in otoliths is still relatively new analytical technique and time consuming. We provided this analysis as a proof of concept for future ecological applications.
