# Supplementary material for: Biogeochemical processes create distinct isotopic fingerprints to track floodplain rearing of juvenile salmon
Source: PLoS One. 2021 Oct 28;16(10):e0257444. doi: 10.1371/journal.pone.0257444 (PMC8553044; doi:10.1371/journal.pone.0257444)
Supplement: Attachment — Submitted filename: Reviewer_response_2021-05-28.docx [file pone.0257444.s004.docx]

To whom it may concern,

I am pleased to resubmit this following manuscript PONE-D-20-24634R1. I appreciate the time and thought of the reviewer comments on the revised manuscript and I feel that this improved the manuscript yet again. I am including the response to comments below as well as a “clean” and “marked up” version of the manuscript and updated figures that incorporates comments and suggestions from the reviewer. I hope that this updated version of the manuscript meets the expectation of the reviewer and the editor. I am looking forward to hearing your thoughts on the revised manuscript.

Thank you for your time,

Carson Jeffres

Reviewer #2:

The authors did a nice job of addressing the previous comments and this version of the manuscript is significantly improved. There is still an issue with a lack of clarity in the description of methods and results for the mixed effect model (described below) that should be addressed. Additionally, there are some important features of the study that could influence interpretation of the findings that should be addressed specifically in the discussion including the caging effect on the Sacramento River fish and the low number of otoliths examined.

COMMENT: Mixed model
The authors put a lot of effort into revising the mixed effect models to account for sources of variation in the data. However, the description of the methods and results are still unclear. For example, on lines 270-271 the model is described as applied to stomach contents, but supplemental Table 1 has model results for both stomach contents and muscle tissue. In the main text, there are no mixed effect model results for muscle tissue, only a description of the boxplot (although the discussion mentions muscle tissue on lines 562-564).

Related to this is the LDFA analysis that was applied to stomach contents but not muscle tissue. If both sources of information are available (stomach contents and muscle), why not apply the same statistical models to both? Doing this would provide a nice way to determine how well the chosen stable isotopes can classify rearing location for different assimilation periods (stomach contents=source, fish=time integrated assimilation, otolith=permanent record). If muscle tissue results are not analyzed and reported in the same way as the stomach contents, some justification for excluding it should be provided.

RESPONSE:

We have clarified language in this section. Namely, all the muscle values have been removed from the mixed effects model (MEM) in the supplemental (Table 1) and removed from the corresponding text. The stated objective of the MEM was to ask the extent to which the CNS values varied between habitats as a function of water year. The diet content of the individual fish is the best source of information for that analysis as it represents the proximate source-diets on the two habitats. The muscle data from the paired fish includes variability due to time spent in the habitats and time integrated assimilation and was therefore deemed not appropriate for the MEM. We have crystalized the data sources and specific statistical analyses used to answer the specific objectives in the first paragraph of the results as a roadmap for the results based on the helpful framework of (stomach contents=source, fish=time integrated assimilation, otolith=permanent record). Due to the different assimilates rates of the different tissues, specific data sources were used to address the individual questions.

COMMENT:
The description of the mixed model results on lines 379-386 is insufficient. The authors describe that all sulfur values in the Yolo Bypass were significantly lower than those in the river but no test statistics or coefficients are provided. Supplementary Table 1 does not provide any test statistics or coefficients for the river floodplain comparison or a description of which model was most parsimonious according to the LR tests. Providing these is important to be able to interpret the results, particularly if findings of significant differences are asserted.
Line279-283: “Caged” was not included as a random effect but were those fish included in the model?

RESPONSE:

We have clarified language in this section and added statistical analysis describing differences in spatial variation in δ¹³C and δ³⁴S. Test statistics are now included in the text (lines 845-901) in the results sections and Supplemental Table 1 has been simplified.

COMMENT:

Discussion
There is a lot of set up in the introduction about the value of otoliths but then only 5 total otoliths (three floodplain and two river) are analyzed with only two of the five reported in the main text. Some discussion of the uncertainty inherent in this low sample size is needed. The caged river fish that otoliths were sampled from had much less trophic variability relative volitionally migrating fish and floodplain fish. Additionally, the variation in sulfur isotope ratios between the river and floodplain was reduced in wet years. Both of these factors could reduce clarity in the archival record of otoliths (and time integrated value of muscle tissue) under natural rather than treatment conditions. The data presented suggests the use of stable isotopes of sulfur to identify floodplain rearing is very promising. An explicit discussion of some of the study caveats and how they might be addressed in future studies would be a nice addition.

RESPONSE:

Sulfur isotopes in otoliths remains more of a boutique assay and remains incredibly time consuming and expensive for ecological/fisheries samples sizes (although see Johnson et al. 2012). The expense of this assay and limited project budget precluded a larger numbers of otoliths to be analyzed and therefore the inclusion of otoliths served more of a proof of concept. This is highlighted in the figure of the two otoliths as “examples” of the two distinct profiles. However, all 5 otoliths were analyzed and discussed in the paper. We have clarified this in the paper. We recognize that as with any small sample size, one risks the samples selected not representing the entire variability exhibited in a population. We have added this as a limitation of the study and suggest further research needed to adequately capture the individual variability that may exist, especially given the variability observed in the 2 wild-caught fish. Since the submission of this manuscript, a complementary paper on the use of fish eye lens isotopes has been published (Bell-Tilcock et al. 2020) and is a much more appropriate tissue to characterize floodplain use as an archival tissue (as lenses are more protein-rich than otoliths) and the analytical throughput is significantly faster as laminae are submitted to traditional stable isotope facilities for analysis. The benefit of the otoliths is that one could link the microstructure to the geochemistry and interpret the number of days fish reared on flooplains whereas the specific time-chronology recorded in an individual fish lens laminae remains uncalibrated but more feasible for ecological and fisheries application. We updated the discussion to include this new research and a eye lens/otolith comparison.
